# A Novel Wedge Anchor System for Double-Layer CFRP Plate Cables: Concept, Theoretical Analysis and FEA

**DOI:** 10.3390/ma17143608

**Published:** 2024-07-22

**Authors:** Zeping Zhang, Jie Bai, Qingrui Yue, Guowen Xu, Xiaogang Liu

**Affiliations:** 1 Research Institute of Urbanization and Urban Safety, School of Civil and Resource Engineering, University of Science and Technology Beijing, Beijing 100083, China; zzp_ustb@163.com (Z.Z.); yueqr@vip.163.com (Q.Y.); 2 Research Center of Shanghai Carbon Fiber Composite Application Technology in Civil Engineering, China Construction Eighth Engineering Division Co., Ltd., Shanghai 200122, China; whitecarol@foxmail.com (J.B.); xuguow@163.com (G.X.)

**Keywords:** CFRP plate, wedge anchorage, theoretical analysis, finite element analysis

## Abstract

This study introduces an innovative wedge anchor for double-layer carbon fiber reinforced polymer (CFRP) plate cable to address the current limitation of traditional wedge anchors. By employing the design concept of “secondary force transmission path”, the friction force for anchoring the CFRP plate is effectively transferred into the barrel through its contracting wedge, thus reducing the clamping pressure requirement of traditional wedge anchorage for anchoring thick or double-layer CFRP plates. Based on this conception, this study presents a theoretical analysis model for predicting the influence of parameter variations on the compressive stress of the CFRP plate, which can serve as a tool for rapid configuration preliminary design. Through finite element analysis, the internal stress distribution of the anchor is thoroughly investigated, and the theoretical analysis model for fast predicting compressive stress of CFRP plate is also validated. The results also indicate that the anchorage conception is valid and effective, providing sufficient anchorage of CFPR plates with an anchorage length of 100 mm.

## 1. Introduction

Over the past three decades, the use of carbon fiber reinforced polymer (CFRP) composite materials in fabricating cables as a substitute for steel cables has emerged as a primary research focus in the field of high-performance cable structures [1]. This design concept stems from the outstanding properties of CFRP itself. Compared to steel, CFRP shows higher strength and lighter weight [2], and its excellent corrosion and fatigue resistance contribute fundamentally to extending the service life of the cables [3,4]. Theoretically, replacing steel with CFRP in cable fabrication addresses significant issues associated with traditional steel cables and significantly advances the development of cable structures. Currently, CFRP tendons and plates represent the primary structural forms used in the production of cables. When the strength is equivalent, CFRP plates with a rectangular cross-section exhibit a larger anchorage area and better bending performance [5]. Therefore, CFRP plates show enormous potential for development in cable structures. A literature review indicates that cable roofs, due to their orthogonal loading characteristics, are considered ideal structural applications for CFRP plate cables [1,6]. However, this novel CFRP cable roof structure also poses a series of challenges for the anchorage of CFRP plates.

The influence of the new CFRP cable roof structure system on anchor design manifests mainly in two aspects. Firstly, consideration needs to be given to the dimensions of the anchors. Anchors connect the CFRP cables to the supporting framework or other components [7], and they are tailored to suit the structural characteristics of CFRP. For CFRP plates, prestressed structures usually employ both adhesive and mechanical anchors [8]. In cable roof structures, bulky adhesive anchors disrupt the spatial arrangement of CFRP cables and add to the burden of connections [9], conflicting with lightweight design principles.

Additionally, cable roof structures frequently utilize modular design and prefabricated components, expediting construction completion. Conversely, the maintenance period for adhesive anchors is typically longer, thus not conducive to reducing the construction period. Therefore, mechanical anchors, such as the large-angle wedge anchors proposed by Han et al. [10] and designed for beam string structures, are more suitable for CFRP cable roof structures due to their small size, lightweight, and easy installation [1]. These anchors are used to anchor transversely enhanced CFRP tendons, offering convenience in disassembly and reusability. Mohee FM et al. [11] have set clear design criteria for the dimensions and quality of the anchors in designing a small-sized mechanical anchor for anchoring a single layer of 50 mm × 1.2 mm CFRP plate in prestressed structures. Specifically, the anchor’s weight should be less than 7 kg, and the anchorage length should be less than 110 mm for ease of application. Secondly, the quantity of anchors needs to be taken into account. The complex spatial arrangement of cable members in cable roof structures necessitates a significant number of anchors to connect them to the supporting framework effectively. Liu et al. [12] suggested using a more flexible CFRP continuous strip as the prestressing system on the roof. Wrapping CFRP continuous strips at the nodes minimizes the need for anchors, placing them only at both ends. However, this approach may lead to structural instability. Wang et al. [9] proposed a novel integrated barrel-wedge anchorage system (NISWAS) and validated its design concept through finite element analysis (FEA). The system comprised a steel barrel, a multi-channel integrated wedge, and six CFRP tendons with a diameter of 5 mm. Compared to a traditional wedge anchorage system, NISWAS achieves the anchoring of multiple CFRP tendons, significantly improving anchoring efficiency while reducing the volume and self-weight of the anchoring end. This provides a valuable reference for the anchoring of CFRP plates. Therefore, it is necessary to design small-sized mechanical anchors that can simultaneously anchor multiple layers of CFRP plates.

The anchoring principle of mechanical anchors relies on the frictional force generated between the inner surface of the anchor and the interface of the CFRP plate to balance the tension force. Therefore, pressure must be applied perpendicularly to the surface of the CFRP plate [13]. Based on the method of pressure generation, mechanical anchors can be subdivided into bolt-clamping and wedge anchoring, each with unique characteristics and application scenarios. Bolt clamping anchoring features precise pressure control and ease of disassembly, making it suitable for the modular and cyclical construction of cable-stayed roof structures. However, the adverse effects of bolt relaxation on the long-term service performance of anchors must be considered. This may lead to pressure loss on the CFRP plate, making bolt clamping anchoring unable to provide sufficient load-bearing capacity. In contrast, with their special design, wedge anchors exert stronger extrusion pressure during anchoring through the follow-up of the wedge, forming a self-anchor. This characteristic makes wedge anchors excel in long-term fixation scenarios, and higher structural stability requirements, but traditional wedge anchors are unable to anchor multiple-layer CFRP plates. Therefore, considering the impact of bolt relaxation [14], the type of small mechanical anchor studied in this paper is a wedge anchor.

**Figure 1:**
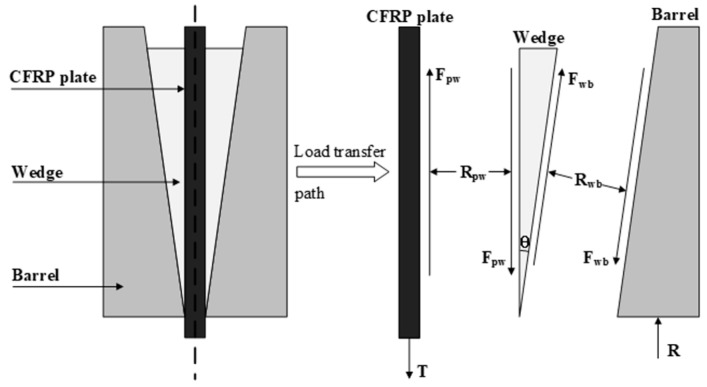
Frictional force transmission path of traditional wedge anchor [15].

Currently, almost all literature on wedge anchors is focused on anchoring single-layer CFRP plates. Anchoring multiple-layer CFRP plates poses challenges without increasing the dimensions of the anchor. The fundamental issue lies in the relatively singular frictional force transmission path within the anchor. Taking traditional wedge anchors as an example, the tension force in the cable is balanced by the friction force generated between the wedge and the CFRP plate. This friction force is ultimately transmitted to the barrel through the interface (CFRP plate-wedge-barrel) and balanced by the restraining force acting on the barrel, as illustrated in Figure 1 [15]. Given the structural form of the CFRP plate, the positive pressure generating the friction force primarily acts on the two wide faces of the CFRP plate. Without increasing the anchoring size, the pressure required for anchoring even the double-layer CFRP plates exceeds the allowable compressive stress range of the CFRP plate. Another potential issue is the uneven distribution of compressive stress on the CFRP plate, which typically increases gradually from the free end to the loading end of the anchor, reaching its maximum at the tip of the wedge [16]. This stress distribution results in the loading end region becoming the primary contribution area to the anchoring capacity. Consequently, the CFRP plate section at the loading end of the anchor also becomes the most susceptible to damage, bearing the maximum compressive stress. This compressive stress concentration may lead to premature failure of the CFRP plate before reaching its ultimate load-bearing capacity. Anchoring a multiple-layer CFRP plate is likely to further amplify this stress concentration phenomenon since the pressure requirements for anchoring multiple layers are much higher than those for anchoring a single layer, and the distance the wedge needs to advance will be greater. Therefore, reducing the pressure requirements for anchoring multiple-layer CFRP plates is crucial. This necessitates the release of a portion of the friction force used to balance external loads onto the barrel through new force transmission paths in advance.

Furthermore, the contribution of relatively low compressive stress in the free-end region to anchoring capacity is minimal [17]. To overcome this issue, Mohee FM et al. [11] proposed a circular edge wedge design concept, which employed a segment of a circular arc with a radius of 3000 mm as the longitudinal profile for the inner surface of the barrel and the outer surface of the wedge. This design ensures a relatively uniform distribution of compressive stress on the CFRP plate, resulting in satisfactory outcomes. Portnov et al. [18] designed special profiled clamping plates with varying curvature surfaces to achieve a smooth transition of shear stress from the loading end to the free end. It is evident that stress concentration within the anchor can be alleviated by designing the contact surface profiles of the wedge, clip, and CFRP plate [1,13]. However, the effects of these designs need to be evaluated through theoretical analysis, as there is currently a lack of effective means to directly measure the internal pressure distribution of the anchor. Overall, these two main issues mentioned above make it difficult for traditional wedge anchors to effectively anchor multi-layer CFRP plates.

To address the current limitation of wedge anchors, which can only anchor single-layer CFRP plate cable, this study aims to achieve efficient anchoring of double-layer CFRP plate cable by proposing an innovative wedge anchor. This paper elaborates on the design concept and methodology of the “secondary force transmission path,” which effectively releases frictional force onto the barrel in advance, thus reducing the pressure requirements for anchoring double-layer CFRP plates. Additionally, this paper presents a theoretical analysis model for calculating the internal contact pressure distribution of the anchor. This model is utilized to predict the influence of parameter variations on the compressive stress of the CFRP plate. Through FEA, the internal stress distribution of the anchor is thoroughly investigated, and the results are compared with the calculations from the theoretical analysis model to validate its effectiveness. The research findings demonstrate that under a 100 mm anchorage length, the new anchor design efficiently anchors double-layer CFRP plate cables with cross-sectional dimensions of 50 mm × 2 mm.

## 2. New Anchor Design Concept

**Figure 2:**
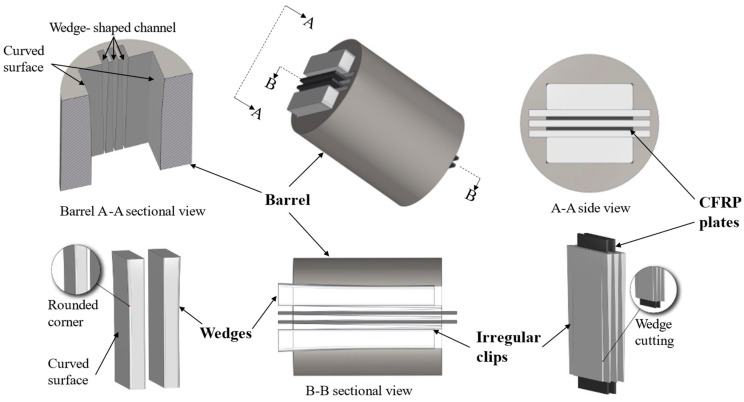
Variable curvature wedge anchor for anchoring double-layer CFRP plates.

The core idea of the new anchor lies in establishing a novel frictional force transmission path to reduce the pressure requirements for anchoring double-layer CFRP plates. Additionally, it ensures a reasonable distribution of pressure on the CFRP plate to avoid stress concentration at the loading end of the anchor. Therefore, two key design concepts for the new anchor are proposed, and their design schemes are illustrated in Figure 2. The first concept pertains to the design of the frictional force transmission pathway. To transmit the frictional force between the double-layer CFRP plates to the barrel through alternative paths, clips need to be introduced between the CFRP plates, connecting them to the barrel, thus forming a secondary force transmission path. For this purpose, a specially shaped clip comprising a flat portion and a wedge portion was designed. It can be visualized as if a rectangular metal clip is cut from both ends along its length at a specific distance. The flat portion of the clip clamps the CFRP plate, while the wedge portion connects to the barrel. The connection between the clip and the anchor is established via a wedge-shaped channel inside the barrel, positioned on the side of the barrel’s inner wall, not under pressure. This channel runs from the loading end to the free end of the barrel, matching the angle of the wedge portion of the clip for proper positioning. After assembling the CFRP plate, clip, and barrel, a secondary frictional force transmission path is established at the interface of “CFRP plate-clip-barrel”.

The next aspect involves designing the surfaces of the barrel’s inner surface and the wedge’s outer surface. In the new design, the combined thickness of the assembled wedge, clips, and CFRP plate must be greater than the thickness of the barrel opening. This difference in dimensions is known as the interference distance [11]. When the assembly is inserted into the barrel opening, the barrel compresses the outer surface of the wedge, creating pressure due to the interference distance. This pressure is then transmitted through the interface and ultimately distributed onto the CFRP plate. Thus, for a reasonable distribution of pressure on the CFRP plate within the anchorage length, the longitudinal profiles of the wedge and barrel were designed using a quadratic function to describe their surfaces. This function is continuous, which helps to avoid stress discontinuities inside the anchor [19]. Additionally, to enhance the pressure in the free-end region and its contribution to anchoring, the slope and curvature of this function gradually increase from the loading end to the free end of the anchor. Lastly, at the exit position of the anchor loading end, it ensures that the first derivative of the function is equal to zero, guaranteeing that the pressure reaches its minimum at the loading end, thereby avoiding stress concentration.

Combining the two aforementioned design concepts, a variable curvature wedge anchor (VCWA) is proposed for anchoring double-layer CFRP plates (Figure 2). The VCWA primarily consists of four components: a hollow circular-section barrel with multiple channels, two variable curvature wedges, three irregular-shaped clips, and double-layer CFRP plates.

Research by Mohee et al. [11] indicated that wedge anchors with an anchorage length of around 100 mm could effectively anchor CFRP plates. According to the design concept of the new anchor, with the optimization of internal stress in the anchor and minimization of anchorage length as objectives, the decisive factors mainly include the magnitude of the compressive stress acting on the surface of the CFRP plate, the friction coefficient between the metal clip and the surface of the CFRP plate, and the interlaminar shear strength of the CFRP plate. Based on previous research conclusions, the in-plane interlaminar shear strength of CFRP plates can reach 60 MPa [20]. To ensure that interlaminar shear failure of the CFRP plate does not occur due to friction in the anchoring zone, the average frictional stress is conservatively limited to no more than 30 MPa as a design objective. Additionally, previous studies have shown that the friction coefficient between steel plates treated with appropriate surface sandblasting and CFRP plates subjected to a roughening treatment can reach around 0.3 [21]. Therefore, according to the principle of friction, the corresponding compressive stress requirement is 100 MPa. Relative to the transverse compressive strength of the CFRP plate, there is still a considerable margin [22]. Based on the fundamental anchoring principles mentioned above, for a CFRP plate with a cross-section of 50 mm × 2 mm and a strength of 2800 MPa, the minimum anchorage length can be less than 100 mm.

Therefore, aiming for a 100 mm anchorage length as our target and satisfying all the design principles mentioned above, a function is prioritized to describe the surface shape as follows:

(1) $$f(x)=\frac{x^{2}}{100000}-\frac{x}{500}+c$$

where *x* represents the anchorage length, with values ranging from 0 to 100 mm, and the parameter *c* controls the thickness of the wedge. To confirm the performance and feasibility of VCWA, the following two sections will conduct theoretical analysis and finite element analysis on VCWA. These two analytical methods will help verify whether the design of VCWA meets expectations.

## 3. Theoretical Analysis of VCWA

Based on the principle of friction, wedge anchoring systems typically require applying significant compressive stress to the CFRP plate before tensioning to ensure sufficient frictional force to balance the tension. This process is generally achieved by setting a certain presetting distance for the wedge. The key point in anchoring double-layer CFRP plates lies in whether the presetting process can provide sufficient compressive stress while avoiding damaging the CFRP plate. Therefore, the theoretical analysis model in this paper focuses on the magnitude and distribution of contact pressure between the wedge, clip, and CFRP plate [11].

**Figure 3:**
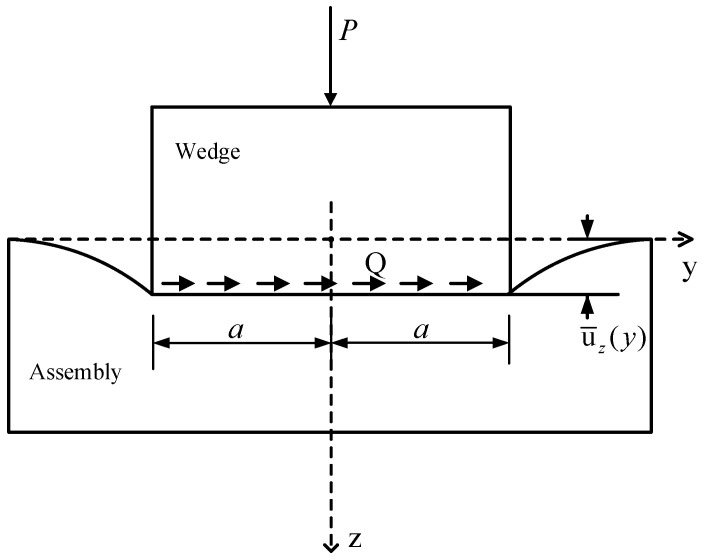
Wedge presses into the Assembly [23].

In this section, the presetting process of the wedge is considered as being acted upon by a rigid flat punch pressing against a plane, as shown in Figure 3. According to previous by Johnson et al. [23], the punch features a flat bottom with a width of 2*a* and sharp square corners, and it is restricted to non-tilted pressing, ensuring that the deformed contact surface remains parallel to the undeformed solid surface. While real wedges cannot be entirely rigid, the compression deformation of the wedge by the barrel serves as the source of anchoring force. Thus, to align the anchor analysis with this theoretical model, several assumptions are proposed [17,24]:

(1) The barrel is considered a rigid body;
(2) Assuming that the deformation of the contact surface between the barrel and the wedge is much smaller than that of the wedge pressing into the clips and CFRP plate assembly;
(3) Spatial issues are simplified to approximate the plane strain problems for analysis;
(4) The assembly of clips and CFRP plate is regarded as an elastic half-space bounded by a flat surface;
(5) Averse frictional forces caused by pressing are neglected.

Now, the assembly consisting of stacked clips and CFRP plates is considered an elastic half-space, and the wedge pressing into this assembly is likened to the action of a rigid flat punch. As shown in Figure 3, the contact interface is the *x*-*y* plane, with the *z*-axis pointing towards the interior of the elastic half-space. *P* represents the pressure exerted by the barrel on the wedge, which causes normal displacement in the *z*-direction. *Q* denotes the tangential force transmitted by the frictional force at the contact interface. Relative to the anchor, transverse frictional forces caused by pressing are typically neglected. In summary, the pressure distribution model inside the anchor is simplified as follows: under given boundary conditions, the pressure distribution at the contact interface caused by the punch pressing into an elastic half-space under the action of pressure *P* is to be determined.

### 3.1. Press-In Caused by Rigid Flat Punch

**Figure 4:**
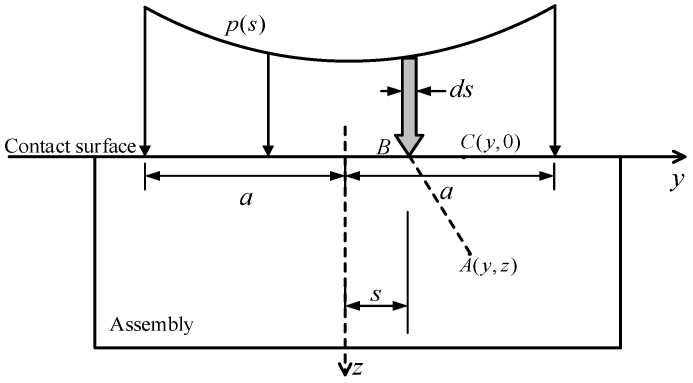
The force analysis of rigid flat punch acting on elastic half-space [23].

According to the research of Johnson et al. [23], the pressing problem of a rigid punch can be regarded as the stress and deformation problem in an elastic half-space caused by a concentrated load of intensity *P* per unit length distributed along the *x*-axis within a narrow band. Combining the assumptions of the above theoretical model, the force analysis is shown in Figure 4. At point *B*, positioned at a horizontal distance *s* from the origin on the contact surface, the force acting on the element of width *ds* can be considered as a concentrated force acting vertically on point *B*, with a value of *pds*. Based on elastic theory, it becomes feasible to ascertain the stress components at any point *A* beneath the elastic half-space induced by these forces, along with the displacement at any point *C* on the contact surface.

For anchorage, it is only necessary to consider the pressure distribution caused by the wedge pressing into the contact surface and the magnitude of compressive stress within the CFRP plate. In the theoretical analysis model, the stress-strain relationship induced by the wedge pressing into the clip and the CFRP plate assembly is linear. This implies that as stress increases, strain increases at the same rate. In this scenario, the clamp and CFRP plate stack are treated as a single entity, and the following are collectively referred to as the assembly. Therefore, after determining the distance *z* of the CFRP plate from the contact surface, the stress component in the *z*-direction at any point within the CFRP plate is given [23]:

(2) $$\sigma_{z}=-\frac{2z^{3}}{\pi }\int_{-a}^{a}\frac{p(s)ds}{{\left[{\left(y-s\right)}^{2}+z^{2}\right]}^{2}}$$

here, *a* represents half of the width of the CFRP plate.

The pressure distribution is given by the integral, as Equation (3) [23]:

(3) $$\int_{-a}^{a}\frac{p(s)}{y-s}ds=-\frac{\pi E}{2(1-\upsilon^{2})}{{\bar{u}}_{z}}^{\prime }(y)$$

where υ represents the Poisson’s ratio of the elastic half-space; ${\bar{u}}_{z}$ denotes the normal displacement of the contact surface; *E* stands for the elastic modulus of the elastic half-space. Since the elastic half-space consists of the clip and CFRP plate, the values of υ and *E* are, respectively, the equivalent Poisson’s ratio and equivalent elastic modulus of the assembly [11]:

(4) $$\upsilon =\frac{\upsilon_{CFRP}t_{CFRP}+\upsilon_{clip}t_{clip}}{t_{total}}$$

(5) $$E=\frac{E_{CFRP}t_{CFRP}+E_{clip}t_{clip}}{t_{total}}$$

where $\upsilon_{CFRP}$ and $\upsilon_{clip}$ represent the Poisson’s ratios of the CFRP plate and the clip, respectively; $E_{CFRP}$ represents the transverse elastic modulus of the CFRP plate; $E_{clip}$ represents the elastic modulus of the clip; $t_{CFRP}$, $t_{clip}$, and $t_{total}$ denote the thicknesses of the CFRP plate, the clip, and the assembly, respectively.

The general solution form of Equation (3) is provided as the following Equation [23]:

(6) $$p(y)=\frac{1}{\pi^{2}{\left(a^{2}-y^{2}\right)}^{1/2}}\int_{-a}^{a}\frac{{\left(a^{2}-s^{2}\right)}^{1/2}g(s)ds}{y-s}+\frac{K}{\pi^{2}{\left(a^{2}-y^{2}\right)}^{1/2}}$$

where g(s) is a known function, and the constant *K* is determined by the total normal load in the contact area, expressed as follows [23]:

(7) $$g(s)=-\frac{\pi E}{2(1-\upsilon^{2})}{{\bar{u}}_{z}}^{\prime }(y)$$

(8) $$K=\pi \int_{-a}^{a}p(s)ds$$

according to the given boundary conditions within the contact area, where q(y)=0, ${\bar{u}}_{z}(y)=\delta_{z}=constant$, Equation (6) can ultimately be simplified to the following [23]:

(9) $$p(y)=\frac{P}{\pi {\left(a^{2}-y^{2}\right)}^{1/2}}$$

Equation (9) allows for the calculation of the compressive stress distribution resulting from the wedge pressing into the assembly in the anchoring width direction. At the edge of the wedge (y=±a), the compressive stress theoretically can reach an infinite value. This presents difficulties when considering the compressive stress near the edge. However, the calculated results from Equation (9) remain meaningful in situations away from the edge, and the compression situation in the middle section of the wedge better reflects the average compressive stress borne by the CFRP plate. Therefore, when validating Equation (9) using FEA, only the middle part is chosen for validation. The boundary where the edge is ignored is determined by comparing FEA results with theoretical results. Subsequently, Equation (9) is substituted into Equation (2) to determine the compressive stress within the compressed object, which is then utilized to calculate the compressive stress within the CFRP plate. These equations are solved using MATLAB software (R2023a).

### 3.2. Contact Pressure Distribution between Barrel and Wedge

Determining the unknown pressure *P* is necessary to calculate the compressive stress distribution using Equation (9). This pressure arises from the squeezing of the barrel on the wedge and is distributed on their contact surface. Therefore, this problem can be treated as a contact problem between the barrel and the wedge. For simplicity, the deformation at the contact is entirely determined by the geometric shapes involved.

**Figure 5:**
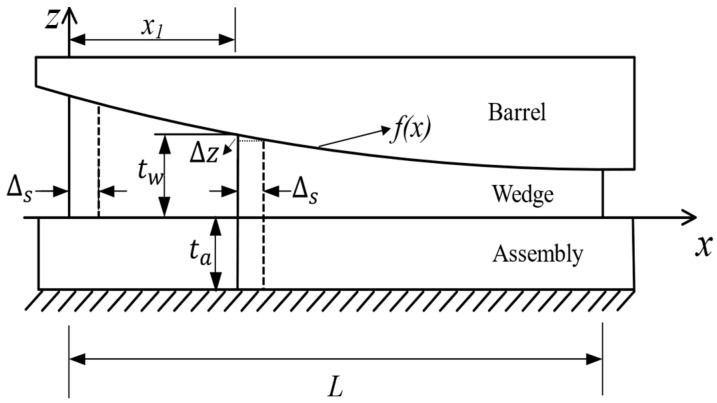
Contact between barrel, wedge and assembly along the anchorage length direction.

Figure 5 illustrates a longitudinal sectional view of the 1/2 model, showing the internal contact within the anchor along the anchorage length direction. Along the *x*-axis, the presetting displacement $\Delta_{s}$ of the wedge will cause the compression deformation of the micro-element at $x_{1}$ in the *z* direction, with the size being as follows.

(10) $$\Delta z=f(x_{1}+\Delta s)-f(x_{1})$$

In the scenario where the barrel is considered a rigid body, the deformation Δz is jointly caused by the deformation of the wedge and the assembly. Thus, the micro-element at $x_{1}$ must satisfy the following conditions:

(11) $$\varepsilon_{W}t_{W}+\varepsilon_{A}t_{A}=\Delta z+\frac{\delta }{2}$$

(12) $$E_{W}\varepsilon_{W}=E_{A}\varepsilon_{A}$$

Here, δ represents the interference distance; $E_{W}$ and $E_{A}$ are the elastic moduli of the wedge and the assembly respectively; $\varepsilon_{W}$ and $\varepsilon_{A}$ are the elastic strains of the wedge and the assembly respectively; $t_{W}$ and $t_{A}$ are the thicknesses of the wedge and the assembly respectively, and since Figure 5 represents a symmetrical structure’s 1/2 model, $t_{A}$ is taken as half of the actual thickness of the assembly.

It is worth noting that within the contact area $0\leq x_{1}\leq L$, when the position of the cross-section at *x*_1_ is different, the thickness of the wedge is also different. Therefore, *t*_w_ is a function of *x*. If the interference distance is entirely controlled by the thickness of the wedge, the expression for *t*_w_ would be:

(13) $$t_{w}(x)=f(x)+\frac{\delta }{2}=\frac{x^{2}}{100000}-\frac{x}{500}+c+\frac{\delta }{2}$$

Similarly, within the contact area $0\leq x_{1}\leq L$, Δz can also be expressed as a function of *x*:

(14) $$\Delta z(x)=\frac{\Delta s^{2}+2x\Delta s}{100000}-\frac{\Delta s}{500}$$

According to Equations (11) and (12), the elastic strain of the wedge can be expressed by the following Equation:

(15) $$\varepsilon_{W}(x)=\frac{\Delta z+\frac{\delta }{2}}{t_{W}+\frac{E_{w}}{E_{A}}t_{A}}$$

Then, the compressive stress in the contact region between the barrel and the wedge is given by the following:

(16) $$\sigma_{W}(x)=E_{W}\varepsilon_{W}=\frac{E_{W}(\Delta z+\frac{\delta }{2})}{t_{W}+\frac{E_{w}}{E_{A}}t_{A}}$$

The load *P* per unit length along the *x*-axis can be expressed as follows:

(17) $$P(x)=2a\cdot \sigma_{W}(x)=\frac{2aE_{W}(\Delta z+\frac{\delta }{2})}{t_{W}+\frac{E_{w}}{E_{A}}t_{A}}$$

It can be observed that the value of *P* is dependent on the position *x* of the section chosen. By substituting Equation (17) into Equation (9), the compressive stress distribution resulting from the wedge pressing into the assembly in the anchorage width direction can be determined.

### 3.3. Parameter Design Scheme

The above theoretical analysis method is used to predict the influence of key parameters on the compressive stress of the CFRP plate. These parameters mainly include the presetting distance of the wedge Δs, the wedge thickness control parameter *c*, and the interference distance δ. For ease of comparison, the section position *x* = 60 is selected, and the initial values of $\Delta_{s}=5$, c=14.1, and δ=0.01 are set. When one of these parameters changes and the other parameters remain at their initial values, then the maximum value given by Equation (2) is computed. Other given parameters are determined based on the dimensions and material properties of the anchor components, with specific values detailed in Table 1. The selection of dimensions and material properties will be discussed in the next section.

**Figure 6:**
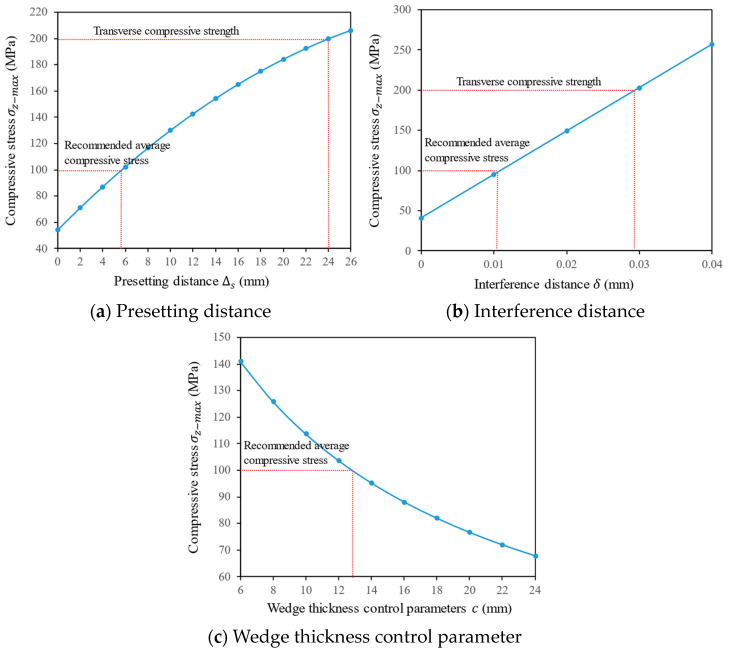
Effect of parameters on compressive stress of CFRP plate.

As shown in Figure 6a, as the presetting distance of the wedge increases, the compressive stress endured by the CFRP plate gradually increases. When $\Delta_{s}=24$ mm, the CFRP plate reaches its transverse compressive strength, and further pushing the wedge may cause damage to the CFRP plate. According to the discussion in Section 2 regarding the anchorage length set to 100 mm in the VCWA and considering a maximum average frictional stress not exceeding 30 MPa with a friction coefficient of 0.3 between the steel plate and the CFRP plate, the average compressive stress requirement for a 50 mm × 2 mm CFRP plate is 100 MPa. Therefore, it is recommended to control the compressive stress of the CFRP plate between 100–120 MPa. Thus, it is suggested that the presetting distance of the wedge be $5.6\leq \Delta_{s}\leq 8.5$ mm. Similarly, considering the relationship between the interference distance and the compressive stress, as shown in Figure 6b, it is recommended to set 0.011≤δ≤0.015 mm. In the VCWA, the thickness of the wedge is the sum of the thickness control parameter *c* and the interference distance δ. Therefore, the value of *c* is related to the thickness of the wedge. As shown in Figure 6c, the compressive stress decreases accordingly as the parameter *c* increases. This trend is consistent with the research conducted by Mohee et al. [11]. on different wedge thicknesses of 6.05 mm, 8.05 mm, 10.05 mm, 13.05 mm, and 15.05 mm, as well as with the research conducted by Ye et al. [26] on wedge thicknesses of 15 mm, 18 mm, and 25 mm. This indicates that as the wedge thickness increases, the maximum contact pressure inside the anchor decreases, and the shear stress on the surface of the CFRP plate decreases as well [11,26]. Based on compressive stress control for the CFRP plate, the value of *c* is determined as 8.9≤c≤12.8 mm. Considering the possibility of increased compressive stress on the CFRP plate due to the wedge’s follow-up during load-bearing, the final optimized choice for *c* is set to 12.8 mm.

Based on the analysis above, a set of proposed optimized parameter design schemes is as follows: presetting distance of the wedge $\Delta_{s}=5.6$ mm, interference distance δ=0.011 mm, and the wedge thickness control parameter *c* = 12.8 mm. In the next section, the FEA of the VCWA will be conducted under these determined parameters to validate their feasibility and further analyze the mechanical behavior within the anchor.

## 4. Finite Element Analysis and Discussion

### 4.1. 3D FE Modeling

**Figure 7:**
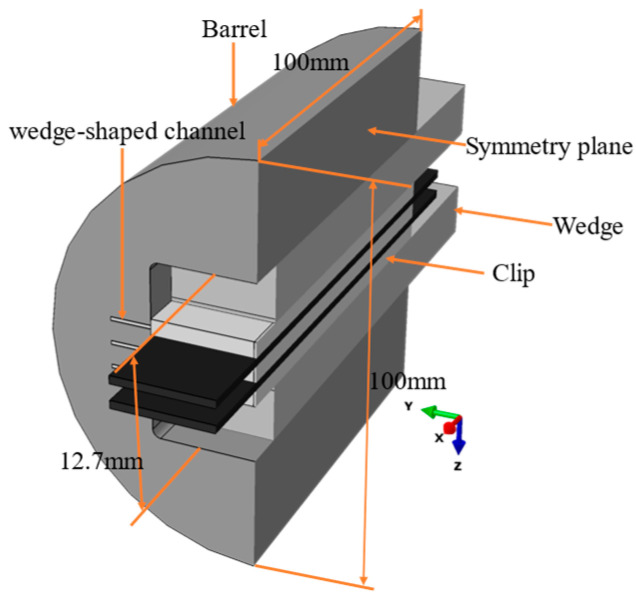
Sketch of the finite element model.

The 3D finite element model of the new anchor is a full-scale model consisting of one barrel, two variable curvature wedges, three special-shaped clips, and two layers of CFRP plates, as shown in Figure 7. Based on the selected key parameters, the dimensions of each component of the anchor are chosen as follows: the barrel is a cylindrical body with a diameter and length of 100 mm each, with an opening size of 12.7 mm at the loading end; the customized clip has a thickness of 4 mm, a width of 50 mm for the flat part, a width of 10 mm for the wedge part, and a cutting angle of 0.92; the CFRP plate has a thickness of 2 mm, a width of 50 mm, and a length of 140 mm, with an additional distance beyond the anchorage length reserved for slip. The *x*-axis is set as the tensile direction of the anchor, parallel to the fiber direction of the CFRP plate, while the *y*-axis and *z*-axis represent the transverse directions of the anchor, perpendicular to the fiber direction of the CFRP plate.

The metal components, such as the barrel, wedges, and clip, are modeled as isotropic elastic-plastic materials. Due to the plastic strengthening of the barrel, wedge, and clip under high-stress conditions, a bilinear hardening model is used to describe their plastic behavior [27]. On the other hand, the CFRP plates are modeled as orthotropic elastic composite materials. Under normal operating conditions, the barrel, wedge, and clip should not undergo plastic deformation. To comply with the assumptions of the theoretical analysis model, materials with higher elastic modulus and yield strength should be selected for the barrel and wedge. Conversely, slightly lower elastic modulus and yield strength materials can be chosen for the clip. Therefore, quenched and hardened alloy steel H13 is chosen for the barrel, alloy steel 40Cr for the wedges, and low alloy steel Q690 for the clip. According to existing research recommendations [19,25], the transverse compressive strength of the CFRP plate is set to 200 MPa, and its longitudinal tensile strength is set to 2800 MPa. The material properties of each component are detailed in Table 1. Based on previous convergence studies and FEA [5,25], a mesh size of 2 mm for the CFRP plates can provide accurate analysis results.

Surface-to-surface contact is utilized to model the contact behavior between the components in this model. Hard contact is used in the normal direction of the contact interface, while a penalty function is introduced in the tangential direction to transmit frictional force. There are four contact interfaces that mainly transfer friction: barrel-wedge, wedge-clip, clip-CFRP plate, and clip-barrel. Literature [21,28,29] indicates that with surface treatments such as sandblasting or adhesive sand, the friction coefficient between steel clips and CFRP plates can range between 0.27 and 0.35. Consequently, the friction coefficient at the CFRP plate-clip interface is set to 0.3. According to the literature [30], the friction coefficient at the wedge-clip interface is set to 0.25 to simulate the significant clamping force between the wedge and the clip. Additionally, the friction coefficient at the barrel-wedge interface is set to 0.07 to simulate the effect of using lubricant at this interface, ensuring that the wedge can enter the barrel and form a self-anchor.

The finite element model incorporates multiple boundary conditions to simulate the pull-out behavior of the new anchor. First, displacement boundary conditions are applied to the barrel surface to restrict its movement in the *x*, *y*, and *z* directions. Meanwhile, the wedges, clip, and CFRP plates experience displacement in the *z* direction during the loading process, thus restricting their movement only in the *y* direction. In the analysis step 1, displacement boundary conditions are applied to the wedges’ cross-section, specifically, the presetting distance, to simulate the presetting process of the wedges. In the analysis step 2, force boundary conditions are applied to the CFRP plates’ cross-section, with tension applied in the *x*-axis direction, and each CFRP plate is set to 2800 MPa. Additionally, the UMAT subroutine based on the three-dimensional Hashin criteria is utilized to evaluate the failure mode of the CFRP plates in both analysis steps.

### 4.2. Stress Distribution of CFRP Plates

**Figure 8:**
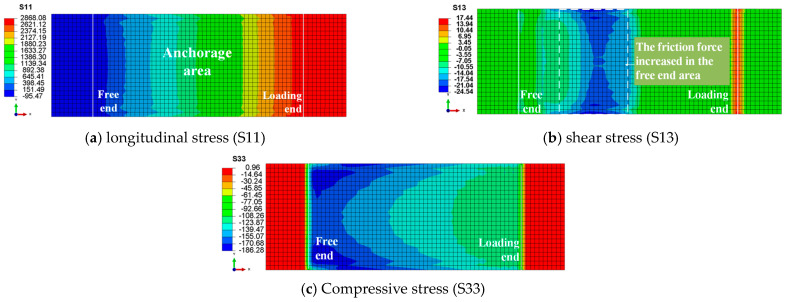
Stress distribution of CFRP plate.

**Figure 9:**
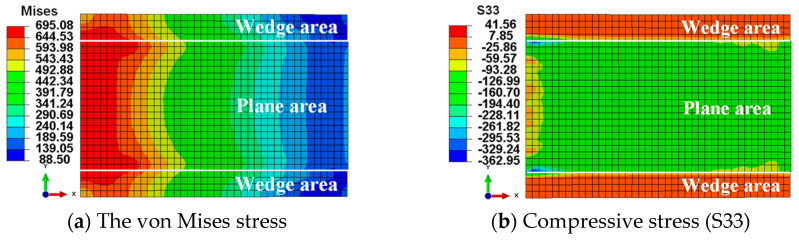
Stress distribution of clip.

As the finite element model is symmetrical, Figure 8 presents only the stress distribution of one CFRP plate. In Figure 8a, the longitudinal tensile stress (S11) in the CFRP plate reaches the ultimate bearing capacity of 2800 MPa, with the maximum tensile stress distributed outside the anchorage area. This suggests that the most probable failure mode of the anchoring system is the rupture of the external tendon, which is an ideal failure mode. In Figure 8b, the shear stress distribution (S13) is shown, caused by the frictional force between the CFRP plate and the clip. Shear stress concentrates at the edges of the CFRP plate, with higher frictional force regions near the free end. This suggests that the free-end region of the new anchor significantly contributes to the anchoring performance. Observing the distribution of compressive stress on the surface (Figure 8c), the variable curvature wedges have essentially fulfilled their initial design purpose, as the compressive stress gradually decreases from the free end to the loading end. In the transverse direction, there is a pattern of higher stress at the edges and lower stress in the middle. Figure 9a reveals that the loading end of the steel clip is more susceptible to plastic deformation than the free end, indirectly indicating that compressive stress concentration has shifted from the loading end to the free end. Figure 9b illustrates that the flat portion of the clip experiences higher compressive stress, whereas the compressive stress in the wedge portion is smaller and distributed more evenly. Since frictional force is directly proportional to pressure and the friction coefficient, the secondary frictional force transmission path of “CFRP plate-clip-barrel” can fulfill its design intent by transferring some of the frictional force to the barrel.

### 4.3. Failure Index Distribution

**Figure 10:**
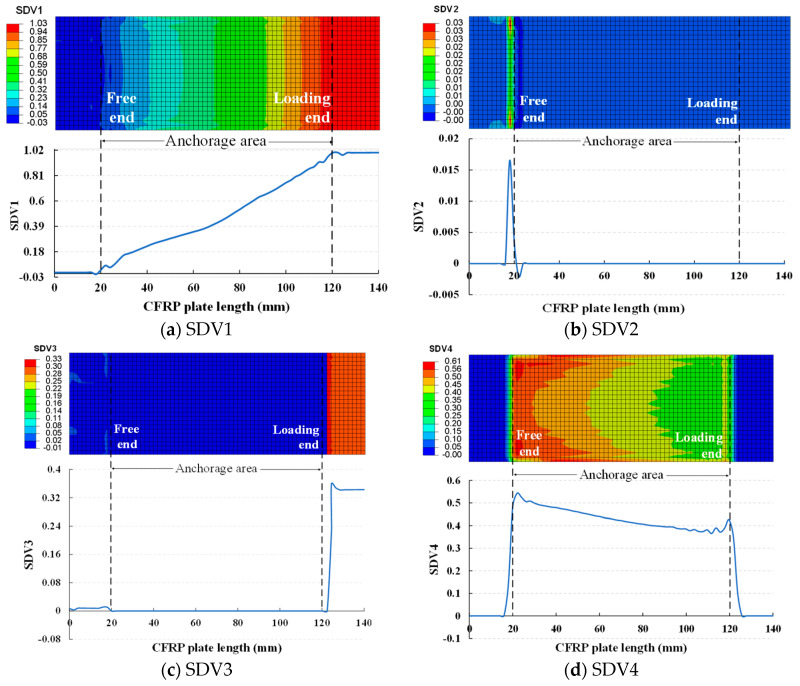
Failure index distribution of CFRP plate.

In this section, the failure index distribution of the CFRP plate based on the three-dimensional Hashin criteria is presented, along with the numerical distribution of each failure index along the length of the CFRP plate, as shown in Figure 10. It is worth noting that the values shown in Figure 10 are from the nodes along the central axis path of the CFRP plate. If the solution-dependent state variables (SDV) value is greater than or equal to 1, the CFRP plate is deemed to have failed. Here, SDV1 represents fiber tensile failure, SDV2 represents fiber compressive failure, SDV3 represents matrix tensile failure, and SDV4 represents matrix compressive failure.

Figure 10a shows that under a tensile load of 2800 MPa, the CFRP plate fails due to fiber tension. However, this failure is limited to the external region of the anchor. Although the failure index gradually increases from the free end to the loading end within the anchoring area, it does not reach 1. Thus, this phenomenon further confirms that the new anchor can fully utilize the performance advantages of the CFRP plate, allowing it to achieve its ultimate bearing capacity. Figure 10b,c reveals that SDV2 and SDV3 exhibit a relatively flat trend throughout the anchoring area. However, SDV2 shows slight fluctuations near the free end, while SDV3 undergoes a sudden change near the loading end. Meanwhile, within the anchoring area, SDV4 gradually decreases from the free end to the loading end. While outside the anchoring area, SDV4 is nearly zero, as depicted in Figure 10d. From the above analysis, it can be observed that the peaks of SDV1 and SDV3 mainly occur near the loading end, while the peaks of SDV2 and SDV4 mainly occur near the free end. If the regions with higher failure indices are considered the most prone to failure in the CFRP plate, the locations prone to failure due to tension are mainly distributed near the loading end, while those prone to failure due to compression are mainly distributed near the free end. This indicates that the new anchor successfully transfers the stress peaks from the loading end to the free end, effectively avoiding the superposition of tensile stress concentration.

### 4.4. Validation of Theoretical Analysis Model

For wedge anchors based on frictional principles, experimental measurement of the contact pressure between the surfaces of internal components is challenging. Hence, mathematical theoretical analysis and finite element methods become the most effective approaches to understanding the distribution of internal pressure in the anchor. To validate the effectiveness of the theoretical analysis model, this section compares the numerical results of the theoretical analysis model with FEA.

#### 4.4.1. Contact Pressure between Barrel and Wedge

**Figure 11:**
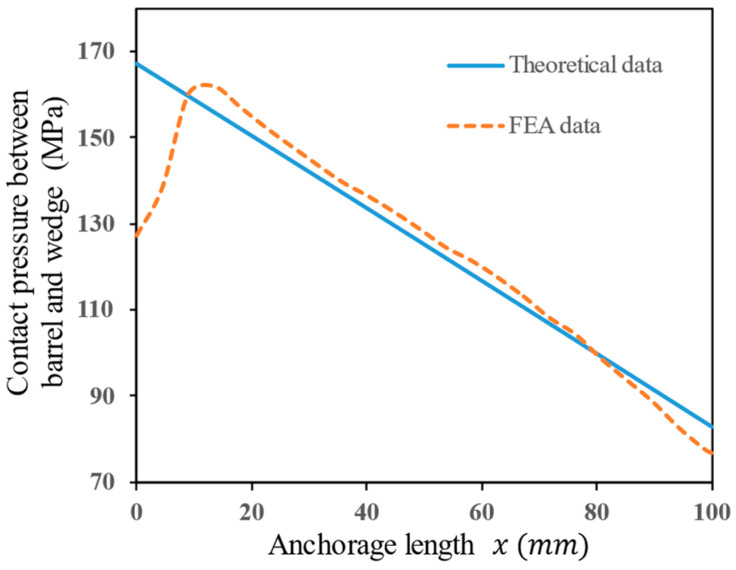
Comparison of theoretical and FEA data on contact pressure between barrel and wedge.

Due to the curved surface design of the inner surface of the barrel and the outer surface of the wedge, the uneven thickness of the wedge along the anchorage length direction causes an uneven distribution of contact pressure between the barrel and the wedge. According to the theoretical calculation from Equation (16), the contact pressure distribution is closely related to the function describing the curved surface design. The intention behind choosing the function is to concentrate the pressure at the loading end of the anchor towards the free end, so the form of the distribution of contact pressure is related to the correctness of the selected function. Figure 11 illustrates the pressure distribution of the contact interface between the barrel and the wedge along the anchorage length direction. Theoretical data are calculated using Equation (16), while the FEA data represents the compressive stress (S33) at the contact interface between the barrel and the wedge. Although there is a discrepancy in the data trend between the theoretical analysis model and the FEA model near the free end, there is good consistency between the FEA numerical analysis results and the theoretical analysis results in other regions. From the free end to the loading end, the pressure distribution along the contact interface between the barrel and the wedge shows a gradual decrease. This form of pressure distribution helps increase the contribution of the free end region to anchoring while effectively avoiding stress concentration at the loading end of the anchor. Therefore, choosing Equation (1) to express the surface shape is justified.

#### 4.4.2. Contact Pressure between Wedge and Assembly

**Figure 12:**
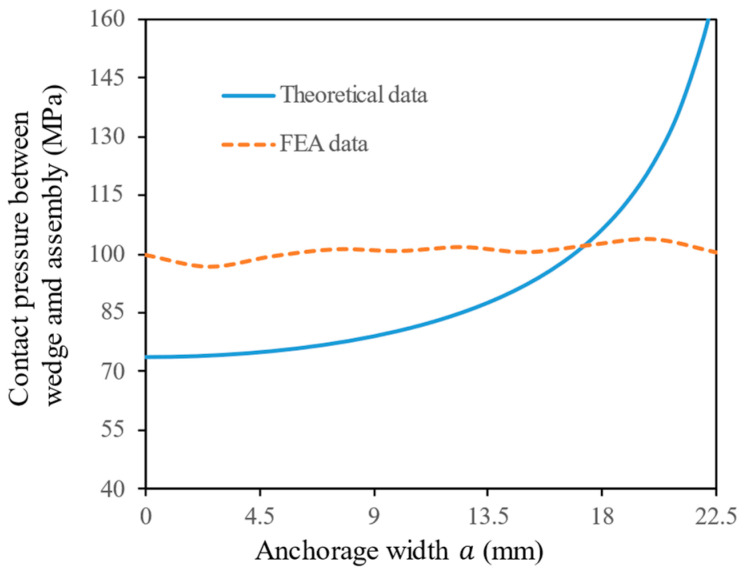
Comparison of theoretical and FEA data on contact pressure between wedge and assembly.

Figure 12 illustrates the distribution of contact pressure between the wedge and the assembly along the anchorage width direction. When a≤18, the contact pressure is relatively uniform, the theoretical data provide a lower envelope in comparison to the FEA data. When a≥18, due to the singularity of Equation (9) at the contact edge, the theoretical value can approach infinity, which is not physically possible. Therefore, the results of the theoretical calculation model beyond the boundary a>22.5 are disregarded. Within the boundary a≤22.5, the average value of the theoretical data has a relative error of 5.7% in comparison to that of the FEA data. In summary, Equation (9) provides accurate predictions of average contact pressure within the contact area a≤22.5. However, when exceeding this area, it is necessary to analyze the results in conjunction with FEA to more accurately describe the contact pressure distribution between the wedge and the assembly.

#### 4.4.3. Compressive Stress of CFRP Plate

**Figure 13:**
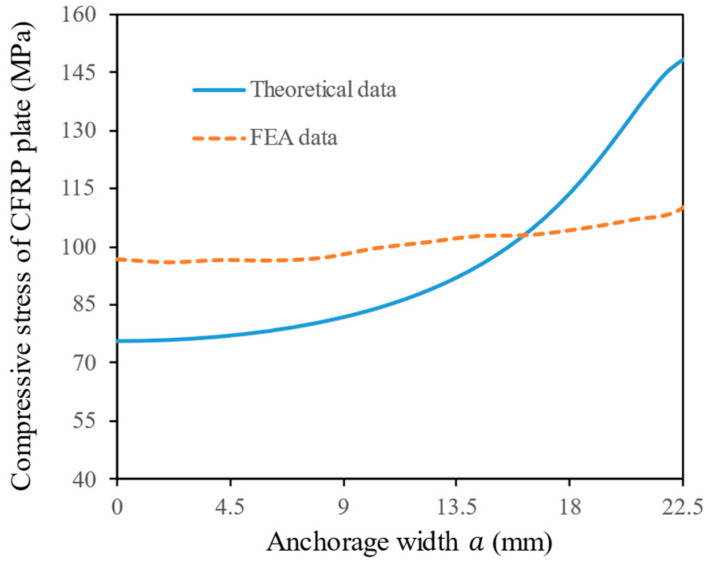
Comparison of theoretical and FEA data on compressive stress of CFRP plate.

Equation (2) represents the internal compressive stress in the CFRP plate, corresponding to the FEA data of S33 in the middle layer of the CFRP plate. Their calculated results are plotted in Figure 13. Along the anchoring width direction, the FEA data show a stable upward trend, reaching maximum values at the edges. The change trend in theoretical data is consistent with FEA data, but it exhibits more drastic fluctuations due to the limitation of the assumptions during theoretical calculation. From Figure 13, it can be observed that when a≤16, the prediction results of the theoretical model are relatively conservative while when a>16, the prediction results of the theoretical data become more aggressive. This is due to the singular solution of the theoretical model at the contact edge, as explained in Section 4.4.1. Because the concentrated force acting on the cross-section of the CFRP plate is constant, the theoretical solution near the contact edge is overestimated, while the theoretical solution in the middle region is underestimated. Nevertheless, excluding the singular region of the theoretical solution at the very edge, the relative error between the average value of the theoretical data and that of the FEA data is 6.8% within the boundary a≤22.5. Therefore, Equation (2) remains useful for rapidly predicting the average compressive stress in the CFRP plate inside the anchor.

## 5. Conclusions

This paper introduces an innovative design concept for wedge anchors intended for double-layer CFRP plate cables. By establishing a theoretical analysis model to calculate the internal compressive stress distribution of the anchor, the basic parameters and design schemes of the new anchor are determined. FEA is conducted to comprehensively analyze the design concept and mechanical performance of the anchor and validate the effectiveness of the theoretical analysis model for predicting compressive stress distribution. The conclusions can be drawn as follows:

(1) Introducing the design concept of a “secondary force transmission path” has successfully reduced the compressive stress demand for anchoring double-layer CFRP plate cables. Simultaneously, the design of the curvature-variable wedge avoids stress concentration at the loading end of the anchorage, addressing the two major issues of “single load transmission path” and “stress concentration at the loading end” in traditional wedge anchors. FEA indicates that the new anchorages can efficiently anchor CFRP plates with a tensile strength of 2800 MPa under the condition of an anchorage length of 100 mm;
(2) Based on a theoretical analysis model of the anchorages proposed by contact mechanics, the calculated average compressive stress on the CFRP plate inside the anchorages corresponds well with FEA results, with a relative error not exceeding 6.8%. This model can be used for rapidly calculating the distribution of contact pressure between internal components of the anchorages, predicting the influence of key design parameters on the compressive stress of CFRP plates, and serving as a simplified theoretical method for preliminary design and parameter optimization of anchoring systems;
(3) Based on the results of the theoretical analysis model and FEA, a set of proposed optimized parameter design schemes is as follows: presetting distance of the wedge Δ_s_ = 5.6 mm, interference distance *δ* = 0.011 mm, and the wedge thickness control parameter *c* = 12.8 mm.

Although the rapid theoretical analysis model proposes optimized parameters of the novel wedge anchorage for double-layer CFRP plate cable, and FEA validation work validates its effectiveness, further experimental investigation is still needed because the actual performance of CFRP cable anchorage will also be influenced by issues such as configuration complexity and fabrication accuracy. The authors are preparing experimental specimens and applying for the relevant patents.

## Figures and Tables

**Table 1:** Mechanical properties of anchor components [19,25].

Part	CFRP Plate	Clip	Wedge	Barrel
Material	CFRP	Q690 steel	40Cr steel	H13
Yield strength, $f_{y}$ (MPa)		690	785	1366
Longitudinal tensile strength, *X*_t_ (MPa)	2800	770	980	1580
Longitudinal compressive strength, *X*_c_ (MPa)	1500			
Transverse tensile strength, *Y*_t_, *Z*_t_ (MPa)	80			
Transverse compressive strength, *Y*_c_, *Z*_c_ (MPa)	200			
Transverse tensile strength, *S*_12_, *S*_13_ (MPa)	120			
Transverse shear strength, *S*_23_ (MPa)	100			
Longitudinal modulus of elasticity, *E*_1_ (MPa)	165,000	206,000	210,000	210,000
Transverse modulus of elasticity, *E*_2_, *E*_3_ (MPa)	9500			
Longitudinal Poisson’s ratio, *ν*_12_, *ν*_13_	0.17	0.3	0.3	0.3
Transverse Poisson’s ratio, *ν*_23_	0.45			
Longitudinal shear modulus, *G*_12_, *G*_13_ (MPa)	5500			81,000
Transverse shear modulus, *G*_23_ (MPa)	3275			
Ultimate strain, *ε*_ult_ (%)	1.7	14	15	9

## Data Availability

The data that support the findings of this study are available from the authors, but restrictions apply to the availability of these data, which also form part of an ongoing study.
